# Double suicide genes selectively kill human umbilical vein endothelial cells

**DOI:** 10.1186/1743-422X-8-74

**Published:** 2011-02-21

**Authors:** Weiguo Jia, Longyong Mei, Yanping Wang, Lunxu Liu, Guowei Che

**Affiliations:** 1 Department of Geriatrics, West China Hospital, Sichuan University, Chengdu 610041, PR China; 2 Department of Thoracic Surgery, West China Hospital, Sichuan University, Chengdu 610041, PR China; 3 The Laboratory of Molecular Diagnosis of Cancer, West China Hospital, Sichuan University, Chengdu 610041, PR China

## Abstract

**Background:**

To construct a recombinant adenovirus containing CDglyTK double suicide genes and evaluate the killing effect of the double suicide genes driven by kinase domain insert containing receptor (KDR) promoter on human umbilical vein endothelial cells.

**Methods:**

Human KDR promoter, *Escherichia coli *(*E. coli*) cytosine deaminase (CD) gene and the herpes simplex virus-thymidine kinase (TK) gene were cloned using polymerase chain reaction (PCR). Plasmid pKDR-CDglyTK was constructed with the KDR promoter and CDglyTK genes. A recombinant adenoviral plasmid AdKDR-CDglyTK was then constructed and transfected into 293 packaging cells to grow and harvest adenoviruses. KDR-expressing human umbilical vein endothelial cells (ECV304) and KDR-negative liver cancer cell line (HepG2) were infected with the recombinant adenoviruses at different multiplicity of infection (MOI). The infection rate was measured by green fluorescent protein (GFP) expression. The infected cells were cultured in culture media containing different concentrations of prodrugs ganciclovir (GCV) and/or 5-fluorocytosine (5-FC). The killing effects were measured using two different methods, i.e. annexin V-FITC staining and terminal transferase-mediated dUTP nick end-labeling (TUNEL) staining.

**Results:**

Recombinant adenoviruses AdKDR-CDglyTK were successfully constructed and they infected ECV304 and HepG2 cells efficiently. The infection rate was dependent on MOI of recombinant adenoviruses. ECV304 cells infected with AdKDR-CDglyTK were highly sensitive to GCV and 5-FC. The cell survival rate was dependent on both the concentration of the prodrugs and the MOI of recombinant adenoviruses. In contrast, there were no killing effects in the HepG2 cells. The combination of two prodrugs was much more effective in killing ECV304 cells than GCV or 5-FC alone. The growth of transgenic ECV304 cells was suppressed in the presence of prodrugs.

**Conclusion:**

AdKDR-CDglyTK/double prodrog system may be a useful method for suppressing tumor angiogenesis.

## Background

Anti-angiogenic therapy has been proved to be a rational approach in the treatment of solid tumors [1]. The advantages of anti-angiogenic approach include new vessels targeting, the lack of mutations in the endothelial cells, and the amplification effect on tumor killing [2]. But targeting endothelial cells by gene therapy was hampered by the inefficiency of the vascular-specific promoters. In the present work, we used a modified kinase domain insert containing receptor (KDR) promoter to direct gene expression in endothelial cells. KDR gene is strictly expressed only in vascular endothelial cells. The activity of the KDR promoter in endothelial cells is similar to that of the potent SV40 promoter/enhancer and it is specific to endothelial cells, i.e., the activity of KDR promoter in other cell types is markedly diminished [3].

A prerequisite for effective anti-vascular gene therapy is the use of a potent "killer" gene. A suicide gene is a gene encoding an enzyme that converts nontoxic prodrugs into toxic forms [4]. Studies have indicated that apart from direct killing effects of a suicide gene, which kills its host cells only, its "bystander effects" could induce death to the nearby cells. These characteristics allow a therapeutic application of suicide genes to tumors. TK and CD gene are the most widely studied suicide genes [4,5]. But both of them have their drawbacks. Therefore, the fusion gene TKglyCD is proposed to be a new suicide gene with better therapeutic effects [6].

In this study, we proposed that the use of KDR promoter in combination with double suicide genes that were specifically activated in the tumor's vascular endothelial cells would provide us with high levels of tumor specificity. The aim of the present work was to generate a replication-deficient adenovirus vector, expressing TK and CD under the control of KDR promoter, as an anti-angiogenesis tool in cancer therapy.

## Materials and methods

### Cell culture

Human umbilical vein endothelial cells ECV304 and human hepatoma cells HepG2 were cultured in RPMI 1640 medium containing 10% fetal calf serum. Human embryonic kidney 293 cells were cultured in DMEM containing 10% fetal calf serum.

### Construction of the recombinant plasmids

Plasmids pGL3-Basic and pGL3-Control contained the firefly luciferase gene (Promega). pGL3-Basic had no promoter, whereas pGL3-Control was driven by the SV40 promoter and enhancer. The plasmid pSV-βgal (Promega) contained the β-galactosidases gene driven by the SV40 promoter and enhancer. Reporter construct containing fragment of the human KDR promoter region from -125 to +227 (366 bp) was inserted into pGL3-Basic and named pGL3-KDR. KDR promoter region was amplified from the genomic DNA of ECV304 cells by PCR. The primers were: forward 5'-GCT CGA GTT GTT GCT CTG GGA TGT TCT-3' (containing *Nru*I site), and reverse 5'- GAA GCT TGT GCC GGT AGG AGA GGA TAT -3' (containing HindIII site).

AdKDR-CDglyTK plasmid was created as the following: first, CD and TK genes were amplified from the *E.coli *JM109 DNA and the plasmid pcDNA3-TK, respectively. Then, these two DNA fragments were inserted together into pcDNA3 vector to generate pcDNA3-CDglyTK. After digesting KDR fragment and pcDNA3-CDglyTK with *Nru*l and HindIII, these two DNA fragments were ligated together to form pcDNA3-KDR-CDglyTK. Finally, KDR-CDglyTK DNA fragment from pcDNA3-KDR-CDglyTK plasmid was inserted into pAdtrack vector to generate pAdtrack-KDR-CDglyTK plasmid. The pAdtrack-KDR-CDglyTK and pAdEasey-1 plasmids were co-transfacted into *E.coli *BJ5183 using the electroporation method. Thirty two positive colonies were screened and analyzed by restriction enzyme digestion. The PCR primers were: CD (1.3 kb) forward 5'- AAG CTT AGG CTA GCA ATG TCG AAT AAC GCT -3', and reverse 5'- GGA TCC TCC ACG TTT GTA ATC GAT GGC TTC -3'; TK (1.1 kb) forward 5'- GGA TCC GGC GGG GGC GGT GGA GGA GGG GGT ATG GCT TCG TAC -3', and reverse 5'- TCT AGA TTA GTT AGC CTC CCC CAT CTC -3'; β-actin (500 bp) forward 5'GAC TAC CTC ATG AAG ATC 3', and reverse 5'GAT CCA CAT CTG CTG GAA 3'. All constructs were sequenced from the 5' and 3' ends to confirm orientation and sequence.

### Transfections

Two cell types were transfected using Lipofectamine 2000 (Roche, USA) according to manufacturer's protocol. For luciferase assay, 5 × 10^5 ^cells/35-mm plate were seeded and incubated at 37°C in 5% CO2 overnight. Next day, 2 μg of reporter plasmids DNA and 0.5 μg of pSV-βgal were mixed with the Lipofectamine 2000 reagent and added onto the cell monolayer. After incubation for an additional 48 hours, luciferase activity was measured in duplicate for all samples using a TD-20/20 Luminometer (Turmer Designs, Sunnyvale, CA) and the Promega luciferase assay system [7]. Beta-galactosidase activity was assayed as described previously. The pGL3-Control was used as positive control and its luciferase activity was considered 100%. Viral DNAs were transfected in 293 cells to package viruses. Propagations of the recombinant viruses could be visualized by GFP expression under a fluorescence microscope. The viral supernatant was purified in CsCl gradient with ultracentrifuge and the titration of AdKDR-CDglyTK viruses was measured using plaque formation assay. The recombinant viruses were stored at -80°C for use.

### RNA isolation and Reverse Transcription

Total RNA from cells was prepared using Trizol reagent according to the manufacturer's protocol (Invitrogen, Carlsbad, CA). Two micrograms total RNA was reverse transcribed using the High-Capacity cDNA Archive Kit (Applied Biosystems, Foster City, CA, USA) according to the manufacturer's protocol with a minor modification: addition of RNase inhibitor (Applied Biosystems, Foster City, CA, USA) at a final concentration of 1 U/μl. The complete reaction mixes were incubated at 25°C for 10 min and 37°C for 120 min.

### Western blot analysis

Cell extracts (10 ng total protein/lane) were separated by standard sodium dodecyl sulfate-polyacrylamide gel electrophoresis under reducing conditions and transferred to polyvinylidene difluoride membranes using a semidry blotting apparatus. After blocking in 5% nonfat dry milk in Tris-buffered saline (TBS) containing 0.1% Tween 20, the membranes were incubated with primary antibodies (dilution 1:100) for 1 h. The primary antibodies were rabbit polyclonal antibodies against TK and CD, kindly provided by William Summers, Yale University. Following incubation with primary antibodies and three washes, the membranes were incubated with peroxidase-conjugated goat anti-rabbit secondary antibodies at 1:100 dilution for 1 h. After three washes, the specific protein bands were visualized with chemiluminescence reagent using the Western blot chemiluminescence system.

### In vitro cytotoxicity assays

Briefly, cells were infected in suspension for 1 h at different MOIs and dispensed into 96-well, flat-bottom microtiter plates at a concentration of 1 × 10^4 ^cells in 100 μl per well. The cells were incubated with or without GCV (5 μg/ml; Roche, USA) and/or 5-FC (100 μg/ml; Roche, USA) at 37°C in a 5% CO_2 _Incubator. After incubation for 72 h, cell viability was determined by standard MTT assay using Cell Titer 96^® ^Aqueous One Solution Reagent Kit (Promega). For bystander effect assay, the infected cells and uninfected cells were mixed in a 1:1 ratio and seeded into 96-well plates with 1 × 10^4 ^cells in 100 μl per well. Cells were treated and analyzed as described above. The percentage of viable cells was calculated as % viability = (number of viable cells counted/total number of cells counted) × 100%.

### Flow cytometry analysis of apoptotic cells

To determine the effect of recombinant viruses on apoptosis, 1 × 10^5 ^of infected or uninfected cells/well were seeded into 6-well plates. The cells were incubated in the presence or absence of GCV (5 μg/ml) and/or 5-FC (100 μg/ml) at 37°C in a 5% CO_2 _incubator. After incubation for 72 h, cells were stained using Annexin V-FITC Apoptosis Analysis Kit (PharMingen) and subjected to a FACS Calibur flow cytometry (Becton Dickinson, Mountain View, CA) to sort out the Annexin V-FITC stained apoptotic cells. Data were analyzed by CELLQuest software (Becton Dickinson, San Jose, CA). The apoptotic index (AI) was calculated as the percentage of apoptotic cells in the treated cell population minus the percentage of apoptotic cells in the untreated control cell population.

### TUNEL assay

1 × 10^5 ^of cells/well were seeded into 6-well plates. After 72 h treatment with GCV (5 μg/ml) and/or 5-FC (100 μg/ml), cells were trypsinized and fixed on slides followed by staining using In Situ Cell Death Detection Kit (Roche). Apoptotic cells with characteristic nuclear fragmentation (staining green) were counted in 10 randomly chosen fields. The apoptotic index (AI) was calculated by subtracting the percentage of apoptotic cells in the untreated control cell population from percentage of apoptotic cells in the treated cell population.

### Statistical analysis

For flow cytometry analysis, each experiment was duplicated and repeated three times. For cell viability assay, each experiment was analyzed in triplicate and repeated at least three times. Data were analyzed using One-Way ANOVA and LSD test. A p-value < 0.05 was considered statistically significant.

## Results

### High levels of CD and TK expression induced by the KDR promoter was found in ECV304 endothelial cells, but not in hepatoma HepG2 cells

**Figure 1:**
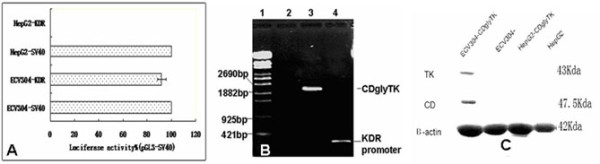
**High level activity of the KDR promoter is specific to endothelial cells**. **A**. The luciferase reporter construct pGL3-KDR was transfected into ECV304 and HepG2 cells. The transfection efficiency was corrected by cotransfection with pSV-βgal. Results are expressed as a percentage of pGL3-SV40 Control activity for each cell type. **B**. The amplification of KDR promoter and CDglyTK fusion gene from recombinant viruses. 1: λ-EclT14I DNA marker; 2: Negative control; 3 and 4: The PCR products of CDglyTK gene and KDR promoter using the recombinant viruses as the template. **C**. Expression of recombinant TK and CD protein in the infected and uninfected cells analyzed by Western blotting.

To determine whether KDR promoter confers endothelial cell-specific expression in cultured cells, we constructed a pGL3-KDR luciferase reporter plasmid. These plasmids were co-transfected into ECV304 endothelial cells and HepG2 cells with pSV-βgal to correct for differences in transfection efficiency. The luciferase activity was normalized to that of the pGL3-Control vector driven by the SV40 promoter/enhancer. We found that the pGL3-KDR luciferase activity was about 92% of that of pGL3-Control vector in ECV endothelial cells (p < 0.05). In contrast, the pGL3-KDR luciferase activity was barely detectable in the HepG2 cells, though the pGL3-Control vector showed high luciferase activity in this cell line (Figure 1A).

### Recombinant viruses

The pAdtract-KDR-CDglyTK and pAdEasey-1 plasmids were co-transfected into 293 cells to package viruses. Most of 293 cells expressed GFP protein after incubation for 3 days, indicating high transfection efficiency. The titer of purified recombinant viruses was measured to be 3 × 10^12 ^pfu/L by plaque formation assay. In addition, we confirmed the presence of the CDglyTK fusion gene and KDR promoter from the boiled recombinant viruses using PCR analysis (Figure 1B).

### Expression of CDglyTK fusion gene

Human umbilical vein endothelial cells (ECV304) and human hepatoma cells (HepG2) were infected with different MOIs of purified recombinant viruses. MOI of 1 only achieved infection of a minority of cells and MOIs of 100 and 200 achieved infection of 94% to 100% of the cells. To identify expression of CDglyTK gene in the infected cells, RNAs and protein were extracted from the infected and uninfected cells and analyzed by RT-PCR and Western blot analysis. The expression of β-actin gene was used as an internal standard. We found that the CDglyTK fusion gene was only expressed in the infected ECV304 cells, but not in the infected HepG2 or uninfected ECV304 or HepG2 cells (Figure 1C).

### Cytotoxicity in vitro

**Figure 2:**
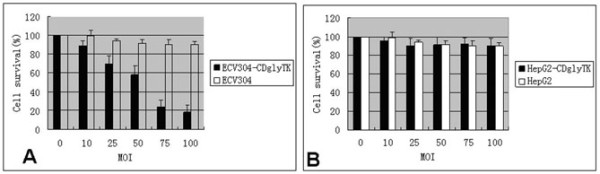
**Cytotoxicity is dependent on MOI**. ECV304 (A) and HepG2 (B) cells at increasing MOI were exposed in vitro to Ad-KDR-CDglyTK in the presence or absence of GCV + 5-FC, and the number of viable cells was determined by MTT methods after 6 days treatment.

To determine if positive or negative KDR expression caused cell death by AdKDR-CDglyTK, ECV304 and HepG2 cells were infected with AdKDR-CDglyTK in the presence or absence of GCV(5 μg/ml) + 5-FC (100 μg/ml). After 6 days of infection, cell death was induced in ECV304-KDR-CDglyTK cells but not in ECV304, HepG2-KDR-CDglyTK, or HepG2 cells. To show if these effects were MOI dependent, ECV304 cells were infected with increasing MOI from 0 to 100. AdKDR-CDglyTK in combination with constant dosage of GCV + 5-FC resulted in MOI-dependent cell death, with an 88% cell death rate at a MOI of 100 (p < 0.05) (Figure 2A). In contrast, HepG2 cells did not show significant cell death (p > 0.05) (Figure 2B).

**Figure 3:**
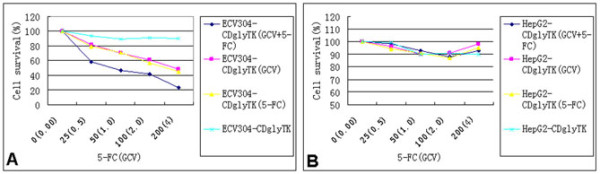
**Cytotoxicity is dependent on concentration of the prodrogs**. Sensitivity of ECV304-KDR-CDglyTK, HepG2-KDR-CDglyTK, ECV304 and HepG2 cells to GCV or 5-FC or both of them.

To determine if the cytotoxicity of the prodrugs was dose-dependent, ECV304 and HepG2 cells were infected with AdKDR-CDglyTK at a MOI of 100. The cells were treated with increasing concentrations of GCV and/or 5-FC for 3 days. We found that KDR-expressing ECV304 cells were highly sensitive to the prodrugs. The cell survival rate decreased significantly along with increasing concentrations of the prodrugs (p < 0.05) (Figure 3A). The cell survival rate of ECV304/KDRp-CD-TK cells treated with 5-FC + GCV was significantly lower than those of the cells treated with 5-FC or GCV alone (p < 0.01). In contrast, HepG2 cells did not show any significant decrease in cell survival after similar treatment (p > 0.05) (Figure 3B).

### Bystander effect

**Figure 4:**
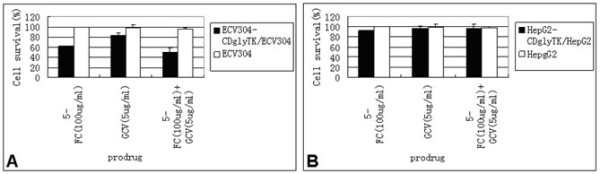
**Bystander effects of 5-FC and/or GCV on ECV304-KDR-CDglyTK/ECV304 and HepG2-KDR-CDglyTK/HepG2 cell death**.

When the ECV304-KDRp-CDglyTK cells were mixed with non-infected ECV304 cells at a 1:1 ratio, the cell death rate was 51.3 ± 3.4% under 5-FC treatment, 46.6 ± 2.3% under GCV treatment, and 74.2 ± 4.9% under 5-FC + GCV treatment, respectively (Figure 4A). The cell death rate caused by the two prodrugs was significantly higher than that caused by either prodrug alone (p < 0.05). Again, HepG2 cells did not show any significant cell death (p > 0.05, compared to the untreated control groups) (Figure 4B).

### AdKDR-CDglyTK/double prodrugs induced apoptosis

**Figure 5:**
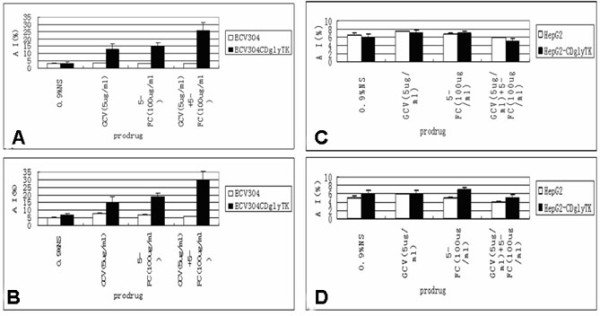
**The cells were killed via apoptosis**. A and C. The ECV304-KDR-CDglyTK, ECV304, HepG2-KDR-CDglyTK, and HepG2 cells were infected with Ad-KDR-CDglyTK at a MOI of 100 and treated with GCV (5 μg/ml) and/or 5-FC (100 μg/ml) for 6 days. Apoptosis was analyzed by Annexin V-FITC staining and flow cytometry analysis. B and D. ECV304 and HepG2 cells infected with Ad-KDR-CDglyTK at a MOI 100 in the presence or absence of o GCV (5 μg/ml) and/or 5-FC (100 μg/ml). Apoptosis was analyzed by TUNEL assays.

We found that the ECV304-KDR-CDglyTK treated with two prodrugs had about 26% apoptosis (Figure 5A). The apoptotic index caused by two prodrugs was significantly higher than that of either prodrug alone (p < 0.05). Again, approximately 6% of apoptotic cells were found in the HepG2 cells treated or untreated with the AdKDR-CDglyTK/double prodrugs (p > 0.05) (Figure 5C). Similar results were obtained when the cells were analyzed with TUNEL assay (Figure B, D).

## Discussion

Angiogenesis is crucial for the progression and metastasis of solid tumor and the vasculature of tumor tissue is different from normal vasculature [8]. Tumor vascular targeting therapy could represent an effective therapeutic strategy to suppress both primary tumor growth and tumor metastasis. A major drawback of current approaches using anti-angiogenesis gene therapy is the lack of tissue-specific targeting. KDR was critically involved in the regulation of angiogenesis, both in young and adult animals [9]. Vascular endothelial cells were renewed at a low speed in normal conditions and their KDR expression levels were very low, whereas tumor vascular endothelial cells proliferated quickly and their KDR expression levels were 500-fold higher than those of the vascular endothelial cells from normal tissues [10]. Therefore, KDR promoter-driven expression of therapeutic genes in tumor vascular endothelial cells can markedly reduce the side effects of gene therapy through targeting the vascular endothelial cells of tumors [11].

There are several advantages that anti-angiogenesis gene therapy may offer: (1) a single vessel supports the survival of many tumor cells by providing oxygen and nutrients. Thus, destroying one vessel may result in the death of many tumor cells [12]; (2) endothelial cells in the tumor vasculature have a lower mutation rate compared to tumor cells, which means that endothelial cells will unlikely acquire resistance to the therapeutic drugs [13]; (3) angiogenesis is infrequent in the adult, which means that therapies which target endothelial cells in tumor vasculature may not damage normal endothelial cells and therefore may have minimal toxicities [13]; (4) one vascular targeting agent may be effective in many solid tumor types; and (5) the endothelial cells are easily accessible by intravenous administration of therapeutic agents [13].

CD and TK are two competent suicide genes. Previous investigations that employed either CD or TK aiming at eliminating tumors have demonstrated limitations. However, the fusion gene of CD and TK presented an exciting superiority in many studies [14-17]. CDglyTK encodes a bifunctional enzyme possessing both CD- and TK-specific activities. Cytotoxicity can be enhanced by concurrently treating CDglyTK-expressing cells with 5-FC and GCV, resulting in a synergistic effect. Another benefit of the fusion gene is its ability to avoid the acquired drug resistance [18,19].

Adenovirus vectors can be prepared at much higher titers than retroviral vectors and have a high efficiency of gene transfer regardless of the proliferative state of tissues whereas retroviral vectors insert their genes only into dividing cells. Although the duration of in vivo gene expression with an adenovirus vector is short, the level of therapeutic gene expression is much higher, which is of much importance in suicide gene therapy because the therapeutic effect of suicide genes is to kill the transgenic cells and cells nearby. A transient and high level expression would be enough, as once its killing effect is realized, the gene expression would be inactivated due to the death of the host cells, thus a long expression duration would be meaningless [20,21].

In our experiment, that the pGL3-KDR luciferase activity was about 92% of that of pGL3-Control vector in ECV304 endothelial cells. Recombinant adenoviruses infected HUVEC cells effectively as 94% cells were infected and expressed GFP when the cells were infected with viruses at a MOI of 100. Transgenic cells were highly sensitive to both 5-FC and GCV, and both prodrugs showed a similar toxicity. However, a marked decrease in cell survival was observed when GCV and 5-FC were used in combination. But our results did not show obvious decrease in the survival rate of HUVEC cells that were infected with recombinant adenoviruses at different MOI, which suggests that the recombinant virus itself is not much toxic to HUVEC cells.

One limitation in our study is that we did not evaluate our system in an in vivo animal study. This in vivo study is ongoing in which the animals bearing lung cancer are treated with different dosages of the recombinant adenovirus, followed by the prodrug treatment. The goal is to test if our system can inhibit tumor angiogenesis thus inhibiting tumor growth in vivo. If this study yields positive results, our next steps will be to conduct human clinical trials, thus translating our laboratory study into clinical applications.

## Conclusions

Our results showed that the adenovirus AdKDR-CDglyTK plus double prodrugs suppressed cell growth and induced apoptosis in ECV304 cells, suggesting that there is some potential in utilizing this system in anti-angiogenesis therapy against tumors.

## Competing interests

The authors declare that they have no competing interests.

## Authors' contributions

JW and GC carried out all the experiments, analyzed results and drafted the manuscript. LL helped to edit the manuscript. LM participated in analysis of data and preparation of the manuscript. YW participated in the design of the study and the critical view of manuscript. All authors read and approved the final manuscript.
